# VA’s implementation of universal screening and evaluation for the suicide risk identification program in November 2020 –Implications for Veterans with prior mental health needs

**DOI:** 10.1371/journal.pone.0283633

**Published:** 2023-04-11

**Authors:** Kritee Gujral, Nazanin Bahraini, Lisa A. Brenner, James Van Campen, Donna M. Zulman, Samantha Illarmo, Todd H. Wagner

**Affiliations:** 1 Health Economics Resource Center, VA Palo Alto Health Care System, Menlo Park, California, United States of America; 2 Center for Innovation to Implementation, VA Palo Alto Health Care System, Menlo Park, California, United States of America; 3 Rocky Mountain Mental Illness Research, Education and Clinical Center, Rocky Mountain Regional VA Medical Center, Aurora, Colorado, United States of America; 4 Department of Psychiatry, University of Colorado, Anschutz School of Medicine, Aurora, CO, United States of America; 5 Department of Physical Medicine and Rehabilitation, University of Colorado, Anschutz School of Medicine, Aurora, Colorado, United States of America; 6 Department of Neurology, University of Colorado, Anschutz School of Medicine, Aurora, Colorado, United States of America; 7 Department of Medicine, Division of Primary Care and Population Health, Stanford University School of Medicine, Stanford, California, United States of America; 8 Department of Surgery, Stanford University School of Medicine, Stanford, California, United States of America; Georgia Southern University, UNITED STATES

## Abstract

**Importance:**

United States Veterans are at higher risk for suicide than non-Veterans. Veterans in rural areas are at higher risk than their urban counterparts. The coronavirus pandemic intensified risk factors for suicide, especially in rural areas.

**Objective:**

To examine associations between Veterans Health Administration’s (VA’s) universal suicide risk screening, implemented November 2020, and likelihood of Veterans being screened, and receiving follow-up evaluations, as well as post-screening suicidal behavior among patients who used VA mental health services in 2019.

**Methods:**

VA’s Suicide Risk Identification Strategy (Risk ID), implemented October 2018, is a national, standardized process for suicide risk screening and evaluation. In November 2020, VA expanded Risk ID, requiring annual universal suicide screening. As such, we are evaluating outcomes of interest before and after the start of the policy among Veterans who had ≥1 VA mental health care visit in 2019 (n = 1,654,180; rural n = 485,592, urban n = 1,168,588). Regression-adjusted outcomes were compared 6 months pre-universal screening and 6, 12 and 13 months post-universal screening implementation.

**Measures:**

Item-9 on the Patient Health Questionnaire (I-9, VA’s historic suicide screener), Columbia- Suicide Severity Risk Scale (C-SSRS) Screener, VA’s Comprehensive Suicide Risk Evaluation (CSRE), and Suicide Behavior and Overdose Report (SBOR).

**Results:**

12 months post-universal screening implementation, 1.3 million Veterans (80% of the study cohort) were screened or evaluated for suicide risk, with 91% the sub-cohort who had at least one mental health visit in the 12 months post-universal screening implementation period were screened or evaluated. At least 20% of the study cohort was screened outside of mental health care settings. Among Veterans with positive screens, 80% received follow-up CSREs. Covariate-adjusted models indicated that an additional 89,160 Veterans were screened per month via the C-SSRS and an additional 30,106 Veterans/month screened via either C-SSRS or I-9 post-universal screening implementation. Compared to their urban counterparts, 7,720 additional rural Veterans/month were screened via the C-SSRS and 9,226 additional rural Veterans/month were screened via either the C-SSRS or I-9.

**Conclusion:**

VA’s universal screening requirement via VA’s Risk ID program increased screening for suicide risk among Veterans with mental health care needs. A universal approach to screening may be particularly advantageous for rural Veterans, who are typically at higher risk for suicide but have fewer interactions with the health care system, particularly within specialty care settings, due to higher barriers to accessing care. Insights from this program offer valuable insights for health systems nationwide.

## Introduction

Suicide is the 10th leading cause of death in the United States (U.S.), and suicides among U.S. adults increased from 29,580 in 2001 to 45,861 in 2019 [1,2]. Given the lack of improvement in suicide rates in the U.S., the Joint Commission on Accreditation of Healthcare Organizations has been re-evaluating current suicide prevention practices and has been moving towards recommending increased screening for mental health conditions, as well as screening for suicide in primary care integrated settings in recent years [2].

Age- and sex-adjusted suicide rates among U.S. Veterans are consistently about 50% higher than non-Veterans [1,3,4]. Although suicides among Veterans decreased in 2018 and 2019, compared to prior years, approximately 6,000 Veterans die by suicide each year [1]. Veterans Health Administration (VA) patients residing in rural areas have a 20% higher risk of dying by suicide compared to VA patients living in urban areas [5]. Urban-rural disparities in suicide risk persist in part because of growing unemployment and lack of health care resources in rural areas, both of which may have been exacerbated by the COVID-19 pandemic [6–9]. The pandemic also intensified risk factors—social isolation, intimate partner violence, and firearm access, which disproportionately affect rural residents [8,10]. Furthermore, reduced interaction with routine health care during the pandemic limited opportunities to screen and treat patients for suicide risk [8], challenging suicide prevention efforts inside and outside VA.

Suicide prevention is a critical priority for the VA and VA has taken several steps to ensure that Veterans, including those living in rural or underserved areas, have access to adequate mental health care needed to prevent suicides. Early and accurate detection of suicide risk among ***all*** Veterans presenting for care is one critical strategy towards reducing overall Veteran suicides [11]. In November 2020, VA implemented a universal screening requirement, which to our best knowledge, represents the largest suicide screening intervention in the U.S. VA has previously led the way on suicide prevention efforts that are now considered best practices. As such, in light of recent nationwide efforts to prevent suicides via universal suicide screening interventions [12–15], we conducted a descriptive evaluation of VA’s large-scale and nationwide implementation of universal suicide screening focusing specifically on Veterans with mental health needs to offer valuable early baseline data and descriptions for health systems aiming to leverage universal screening to reduce suicide risk among high-risk patients with mental health needs.

We focused on Veteran patients who were already engaged in mental health care in the year prior to assess whether the universal approach to screening added value or would results in supplemented care (e.g., evaluation for those who screened positive) for this group of at-risk Veterans. We also assessed whether the universal requirement could reach rural Veterans who typically face more barriers to accessing care and are at higher risk for suicide. We examined the association between implementation of the universal screening requirement and Veterans’ likelihood to receive a suicide screen, follow-up in-depth suicide evaluation, and the likelihood of reported suicide behavior.

## Intervention

VA implemented the Suicide Risk Identification Strategy (Risk ID) in 2018. Risk ID facilitates identification of suicide risk through two key processes: standardized suicide risk screening and comprehensive evaluation for those with a positive suicide risk screen. Initially, Risk ID (first implemented from October 2018 –November 2020), included three steps: (1) primary screen: item 9 of the Patient Health Questionnaire-9 (I-9), (2) secondary screen: Columbia Suicide Severity Rating Scale (C-SSRS) Screener and (3) Comprehensive Suicide Risk Evaluation (CSRE). Individuals who screen positive at one step move on to the next level of screening or evaluation [16]. Implementation of Risk ID in ambulatory care settings was initially focused on Veterans due for required annual screens for depression and/or posttraumatic stress disorder (PTSD), which accounts for approximately 77% of Veterans receiving VA care [16]. In November 2020, Risk ID was modified and reduced to two steps—the C-SSRS Screener followed by the CSRE (S1 Fig), where CSREs may in some instances be administered as the first step without a C-SSRS Screener (e.g., if clinically indicated). At the same time, in November 2020, the VA implemented a new policy requiring that *all* Veterans receiving VA care were screened at least annually (i.e., universal screening requirement) [17]. Universal screening was designed to increase detection of suicide risk among Veterans receiving care across a wide range of settings (e.g., primary care, specialty care including mental health, audiology) [17].

A recent evaluation of Risk ID in ambulatory care settings showed that a positive C-SSRS Screen was associated with increased mental health follow-up and engagement. Importantly, mental health follow-up and treatment after a positive C-SSRS Screen was higher among patients who were not engaged in mental health care in the year prior to suicide screening, compared to patients who were engaged in mental health care in the year prior [18]. While promising, these findings were focused on Risk ID processes prior to implementation of the universal screening requirement. In order to generate additional evidence to inform VA and other health systems, we examined outcomes associated with Risk ID’s universal screening requirement among those already engaged in mental health care.

## Methods

### Study cohort

The study cohort included rural and urban patients who had ≥1 VA mental health care visit in the year 2019. Data on VA patients and visits were obtained from the VA Corporate Data Warehouse (CDW), a VA electronic health records repository. VA clinic stop codes were used for characterizing mental health outpatient visits (e.g., care visits for depression, PTSD, substance use disorder, bipolar disorder, and other mental health conditions) [19]. Rurality was defined as Rural-Urban Commuting Areas codes other than 1 or 1.1 [20]. Veterans were observed 6 months prior to and 12 and 13 months after universal screening.

### Measures & tools

**Item 9 of the Patient Health Questionnaire 9 (I-9)** was VA’s one-question primary screener, which was replaced by the C-SSRS in November 2020. Item-9 asks patients, “Over the past two weeks, how often have you been bothered by thoughts that you would be better off dead or of hurting yourself in some way?” Possible responses were “Not at all,” “Several days,” “More than half the days,” or “Nearly every day.” A positive screen was any answer other than “Not at all.”

**Columbia Suicide Severity Rating Scale Screener (C-SSRS)** is a 6-item measure, where the first five items assess severity of suicidal ideation, including method, intent, and plan; the final item, comprised of two parts, asks about lifetime and recent suicidal behaviors. All items are answered with “yes” or “no.” A positive C-SSRS screen was defined as a “yes” response to items 3, 4, 5, or 6b. The reliability and validity of the original and screen versions of the C-SSRS has been demonstrated in prior studies [18].

**Comprehensive Suicide Risk Evaluation (CSRE)** is a standardized VA template that assists providers in conducting a comprehensive evaluation of a patient’s level of suicide risk to inform risk management. CSREs include detailed prompts related to suicidal ideation, history of attempts, warning signs, risk factors, protective factors and reasons for living, as well as treatment planning.

**Suicide Behavior and Overdose Report (SBOR)** was developed by VA’s Office of Mental Health and Suicide Prevention to standardize the process of suicidal behavior and overdose reporting for Veterans. SBORs are used nationally as the primary data-collection and surveillance process for Veteran suicidal behaviors and overdoses. Clinical staff are trained and required to submit an SBOR when they learn of suicide attempt, drug overdose event, death by suicide or preparatory suicidal behaviors that occurred in the last 12 months [21–23].

### Data

#### Outcomes

We examined three types of outcomes. First, we measured the monthly likelihood of visits that included C-SSRS. In order to capture suicide screens occurring via the historic primary suicide screen I-9, we also measured the likelihood of suicide screen via either the C-SSRS or I-9. Second, we measured likelihood of CSREs if the C-SSRS was positive. Third, we measured the likelihood of a SBOR.

VA health care utilization data were obtained from CDW. Data on suicide screens, evaluations, and suicide behavior reports were obtained from VA’s Program Evaluation Resource Center (PERC), a VA operational group that tracks performance of VA mental health care [24].

#### Covariates

All models were adjusted for Veterans’ age, sex, race/ethnicity, rurality of residence, number of physical and mental health chronic conditions [19,25–27], indicators for diagnoses of substance use disorder (SUD), PTSD and depression, Nosos score (risk score based on the Medicare risk adjustment with additional covariates for mental health and medication usage) [28], VA priority-based enrollment categories (based on Veterans’ service-connected disabilities and other factors) [19,29], marital status, homelessness, high suicide risk indicator, broadband coverage in patients’ residential zip-codes [30] and monthly COVID-19 cases in patients’ residential counties. We included facility fixed effects to control for any time-invariant facility characteristics; some Veterans use more than one VA facility, in which case we controlled for the closest VA facility that provided specialty care.

Age, sex, race, race/ethnicity, rurality of residence, diagnoses, Nosos score, VA enrollment priority and marital status were obtained from CDW electronic health record data. Distances to patients’ closest facilities were obtained from VA Geospatial Service Support Center. Homelessness was defined using outpatient stop codes indicating use of the VA homeless services and diagnosis codes [19]. Data on county-level COVID-19 cases were obtained from the *New York Times* [31].

#### Statistical analysis

We conducted unadjusted analyses at the person-level and adjusted analyses at the patient-month-level. In person-level analyses, we compared characteristics of rural and urban Veterans. We also reported the number of patients (total, rural, urban) who received at least one suicide screen or evaluation, who were identified at-risk based on either a positive I-9 or positive C-SSRS, who received at least one follow-up evaluation, and who had at least one SBOR in the 6-month period pre-universal screening and in the 12-month period post-universal screening implementation. To examine the role of setting of care where screens were administered, we conducted exploratory sub-analyses for patients who had a mental health visit in the 12-month period post-universal screening implementation and those who did not (See Online Supplement S3 Table for a breakdown by rurality and race).

In patient-month-level analyses, we used a pre-post event study design to examine descriptively the associations between timing of VA’s universal implementation of the C-SSRS suicide screener and the likelihood of Veterans receiving a suicide screen, a follow-up in-depth suicide evaluation or CSRE if the C-SSRS was positive, as well as the likelihood of a SBOR, adjusting for patient covariates. We considered a non-VA control group because this policy was implemented at the same time for all VA patients. However, the effect of COVID-19 on employment and insurance coverage [32] made this difficult. As such, our pre-post analysis compared outcomes for our study cohort pre- and post- November 2020, accounting for differences in patient covariates. A break in the outcomes trend post-universal screening implementation, compared to pre-universal screening would signal attributability of the results to the implementation of VA’s universal screening requirement. The person-month analyses were also helpful for seeing if a follow-up evaluation occurred in the same month as the month in which a patient had a positive screen; for analyzing follow-up evaluations, we restricted the analysis to patient-month observations where there was a positive screen. We presented regression coefficients of interest (plotted in Figs 2 and 3) indicating differences in each month, compared to the baseline month of November 2020. These coefficients reflected associations with VA’s universal screening, each month pre- and post-universal screening implementation, adjusting for covariates (additional method details are provided in S1 File).

We also conducted sensitivity analyses to determine if differences across rural and urban patients were driven by broadband coverage in a patient’s area. As these data were only available for 97% of the study cohort (n = 1,608,523), we conducted separate regression analyses for this subgroup of patients including covariate for broadband coverage by zip code [30]. See S2 Fig for analyses stratified by race, ethnicity, gender and age group.

This study was designated by the VA Office of Rural Health and Office of Mental Health and Suicide Prevention as quality improvement and was exempted from review by the Stanford Institutional Review Board. The study followed the Strengthening the Reporting of Observational Studies in Epidemiology (STROBE) reporting guidelines.

## Results

Tables 1 and 2 present results for the person-level analyses, where Table 1 presents sociodemographic and clinical characteristics of the study cohort (n = 1,654,180), stratified by rural (n = 485,592) and urban (n = 1,168,588) Veterans in VA mental health care and Table 2 presents percentages of the study cohort who had suicide risk screens, evaluations, and indications of suicide-behavior, pre- and post-universal screening implementation. In the 12 months post-universal screening implementation, 80% of the cohort was screened using either the C-SSRS, I-9, or the CSRE, 79% via either the C-SSRS or I-9 and 76% via the C-SSRS. Among Veterans with positive C-SSRSs, 80% received follow-up CSREs within the same month. In the 6-month period prior to universal screening, 3.2% of the cohort had at least one positive suicide screen, and 6 months post- and 12-months post-universal screening implementation, 2.9% and 5.1% of the study cohort, respectively, had at least one positive suicide screen.

**Table 1:** Socio-demographic and clinical characteristics of the study cohort, by rurality of residence.

	Rural Veterans	Urban Veterans	P-value^a^
**Sex**			
Male	430,152 (88.6%)	997,029 (85.3%)	<0.001
Female	55,440 (11.4%)	171,559 (14.7%)	<0.001
**Ethnicity**			
Hispanic or Latino	17,371 (3.6%)	110,370 (9.4%)	<0.001
Not Hispanic or Latino	461,206 (95.0%)	1,039,753 (89.0%)	<0.001
Unknown	7,015 (1.4%)	18,465 (1.6%)	<0.001
**Age (in years) [SD]**	58.2 [15.7]	56.1 [16.1]	<0.001
**Homeless**	6,911 (1.4%)	55,445 (4.7%)	<0.001
**Driving distance to primary care facility (in miles)[SD]**	25.5 [18.1]	9.7 [7.0]	<0.001
**Number of Chronic Physical Conditions [SD]**	4.5 [3.1]	4.2 [3.2]	<0.001
**Number of Chronic Mental Conditions [SD]**	1.8 [1.2]	1.8 [1.3]	<0.001
**Diagnosed with Substance Use Disorder in 2019**	83,922 (17.3%)	238,514 (20.4%)	<0.001
**Diagnosed with PTSD in 2019**	222,852 (45.9%)	464,958 (39.8%)	<0.001
**Diagnosed with Depression in 2019**	237,905 (49.0%)	555,232 (47.5%)	<0.001
**Identified as REACH VET** ^b^	16,423 (3.4%)	49,535 (4.2%)	<0.001
**Nosos Score**	1.6 [2.1]	1.7 [2.3]	<0.001
**VA enrollment priority**			
1	286,349 (59.0%)	647,207 (55.4%)	<0.001
2	33,461 (6.9%)	80,377 (6.9%)	0.77
3	42,001 (8.6%)	103,113 (8.8%)	<0.001
4	11,950 (2.5%)	37,196 (3.2%)	<0.001
5	71,452 (14.7%)	198,838 (17.0%)	<0.001
6	8,613 (1.8%)	19,317 (1.7%)	<0.001
7	4,648 (1.0%)	21,871 (1.9%)	<0.001
8	27,118 (5.6%)	60,699 (5.2%)	<0.001
**Race**			
American Indian or Alaska Native	8,878 (1.8%)	15,060 (1.3%)	<0.001
Asian	2,187 (0.5%)	20,391 (1.7%)	<0.001
Black or African American	63,488 (13.1%)	354,217 (30.3%)	<0.001
Native Hawaiian or other Pacific Islander	3,995 (0.8%)	15,089 (1.3%)	<0.001
White	407,044 (83.8%)	763,831 (65.4%)	<0.001
**Marital Status**			
Divorced	116,044 (23.9%)	305,162 (26.1%)	<0.001
Married	266,145 (54.8%)	515,935 (44.2%)	<0.001
Separated	19,033 (3.9%)	57,522 (4.9%)	<0.001
Widowed	16,410 (3.4%)	35,069 (3.0%)	<0.001
Unknown	4,722 (1.0%)	13,676 (1.2%)	<0.001
**Number of observations**	**485,592**	**1,168,588**	

^a^ Differences in percentages of dichotomous variables were tested using the Pearson’s chi-squared test. Differences in means of continuous variables were tested using the two-sample t-test. P-values <0.05 was considered statistically significant.

^b^ VA classifies as REACH VET Veterans who are at increased risk of suicide, based on VA’s predictive model that analyzes existing data from Veterans’ health records to identify statistically-elevated risk for suicide, hospitalization, illness or other adverse outcomes.

**Table 2:** Unadjusted percentage of Veterans in the study cohort with at least 1 VA visit for indicated reasons, pre- and post-universal screening implementation.

**Entire Cohort**					
	**Pre-Universal Screening (May 2020 –Nov. 2020)** ^a^	**6 Months Post-** **(Dec. 2020 –May 2021)**	***p-val*.** ^b^	**12 Months Post-universal screening implementation** **(Dec. 2020 –Nov. 2021)**	***p-val*.** ^b^
Suicide Screen or Evaluation (I-9/C-SSRS/CSRE)	49.6%	58.9%	*<0*.*001*	78.9%	*<0*.*001*
Suicide Screen (either I-9 or C-SSRS)	49.2%	58.7%	*<0*.*001*	78.6%	*<0*.*001*
C-SSRS	25.4%	54.4%	*<0*.*001*	76.1%	*<0*.*001*
Positive Suicide Screen (I-9 or C-SSRS)	3.2%	2.9%	*<0*.*001*	5.1%	*<0*.*001*
C-SSRS Positive	1.2%	1.4%	*<0*.*001*	2.5%	*<0*.*001*
CSRE if C-SSRS+	76.5%	76.2%	*0*.*474*	79.7%	*<0*.*001*
VA SBOR	0.6%	0.5%	*<0*.*001*	0.9%	*<0*.*001*
**Sub-Cohort of Rural Veterans**					
	**Pre-Universal Screening (May 2020 –Nov. 2020)** ^a^	**6 Months Post-universal screening implementation** **(Dec. 2020 –May 2021)**	***P-value*** ^b^	**12 Months Post-universal screening implementation** **(Dec. 2020 –Nov. 2021)**	***P-value*** ^b^
Suicide Screen or Evaluation (I-9/C-SSRS/CSRE)	47.9%	59.7%	*<0*.*001*	79.5%	*<0*.*001*
Suicide Screen (either I-9 or C-SSRS)	47.5%	60.0%	*<0*.*001*	79.4%	*<0*.*001*
C-SSRS	21.6%	55.2%	*<0*.*001*	76.8%	*<0*.*001*
Positive Suicide Screen (I-9 or C-SSRS)	3.0%	2.7%	*<0*.*001*	4.7%	*<0*.*001*
C-SSRS Positive	1.0%	1.3%	*<0*.*001*	2.2%	*<0*.*001*
CSRE if Positive C-SSRS	75.6%	74.3%	*0*.*100*	77.7%	*0*.*004*
VA SBOR	0.5%	0.4%	*<0*.*001*	0.7%	*<0*.*001*
**Sub-Cohort of Urban Veterans**					
	**Pre-Universal Screening (May 2020 –Nov. 2020)** ^a^	**6 Months Post-universal screening implementation** **(Dec. 2020 –May 2021)**	***P-value*** ^b^	**12 Months Post-universal screening implementation** **(Dec. 2020 –Nov. 2021)**	***P-value*** ^b^
Suicide Screen or Evaluation (I-9/C-SSRS/CSRE)	50.3%	58.6%	*<0*.*001*	78.5%	*<0*.*001*
Suicide Screen (either I-9 or C-SSRS)	50.0%	58.4%	*<0*.*001*	78.3%	*<0*.*001*
C-SSRS	27.0%	54.1%	*<0*.*001*	75.8%	*<0*.*001*
Positive Suicide Screen (I-9 or C-SSRS)	3.3%	2.9%	*<0*.*001*	5.2%	*<0*.*001*
C-SSRS Positive	1.3%	1.5%	*<0*.*001*	2.6%	*<0*.*001*
CSRE if Positive C-SSRS	76.8%	76.9%	*0*.*809*	80.4%	*<0*.*001*
VA SBOR	0.6%	0.5%	*<0*.*001*	0.9%	*<0*.*001*

^a^The pre-implementation period includes the implementation month of November 2020, and therefore includes a total of 7 months.

^b^ Differences in pre- vs. post- implementation percentages were tested using the two-sample t-test. P-values <0.05 were considered statistically significant.

In the sub-cohort of Veterans who had a mental health visit in the 12 months post-universal (n = 1,066,334), 91% of Veterans were screened or evaluated via a C-SSRS, I-9 or CSRE (Table 3). In this sub-cohort, 9% (n = 92,501; 6% of the study cohort) were not screened or evaluated for suicide risk despite having had a mental health visit during this time (S3 Table). Rural Veterans were less likely than urban Veterans to have had a mental health visit but not be screened or evaluated for suicide risk (S3 Table).

**Table 3:** Patterns of screening or evaluation by for Veterans who had at least one mental health visit in the post-universal screening implementation period.

Percentage of sub-cohort who had at least one visit with the following screening or evaluation outcome	
Suicide Screen or Evaluation (via either I-9, C-SSRS, or CSRE)	91.3%
Suicide Screen (either I-9 or C-SSRS)	91.1%
C-SSRS	87.7%
C-SSRS Positive	3.7%
*Number of Veterans in this sub-cohort*	*1*,*066*,*334 (64% of the study cohort)*

We also found that 20% of Veterans in our study cohort were screened or evaluated for suicide risk but did not have a mental health visit during the 12 months post-universal screening, with rural Veterans (vs. urban Veterans) who had at least one mental health visit being more likely to be screened or evaluated (S3 Table).

**Fig 1:**
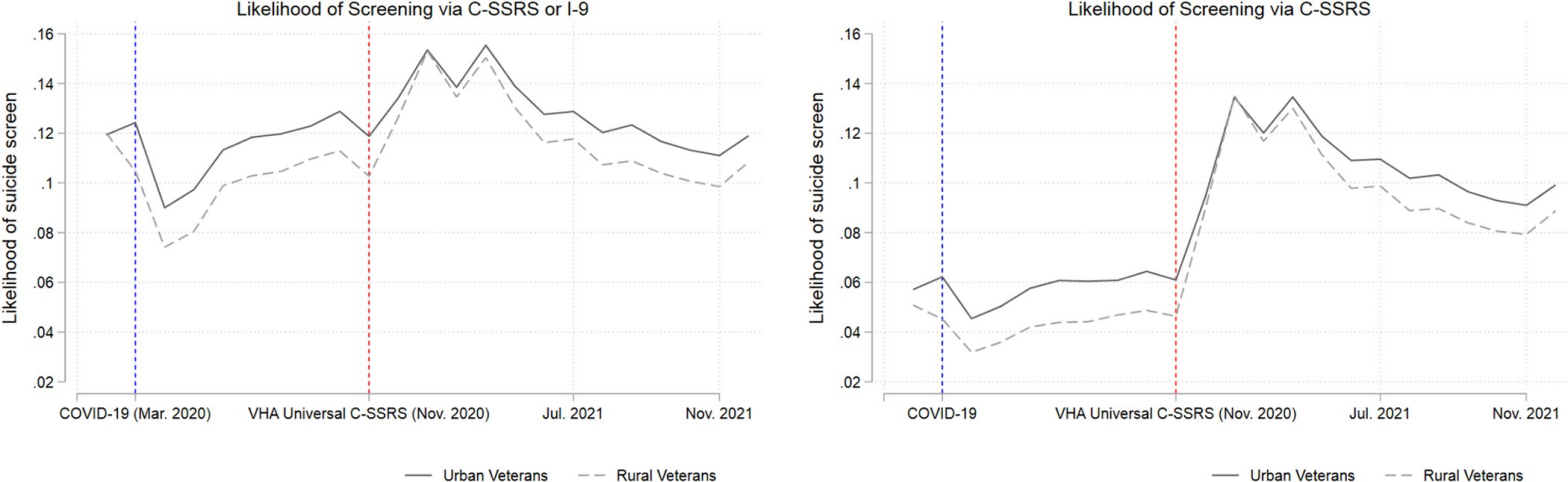
Unadjusted trends in the monthly likelihood of a suicide screen.

**Fig 2:**
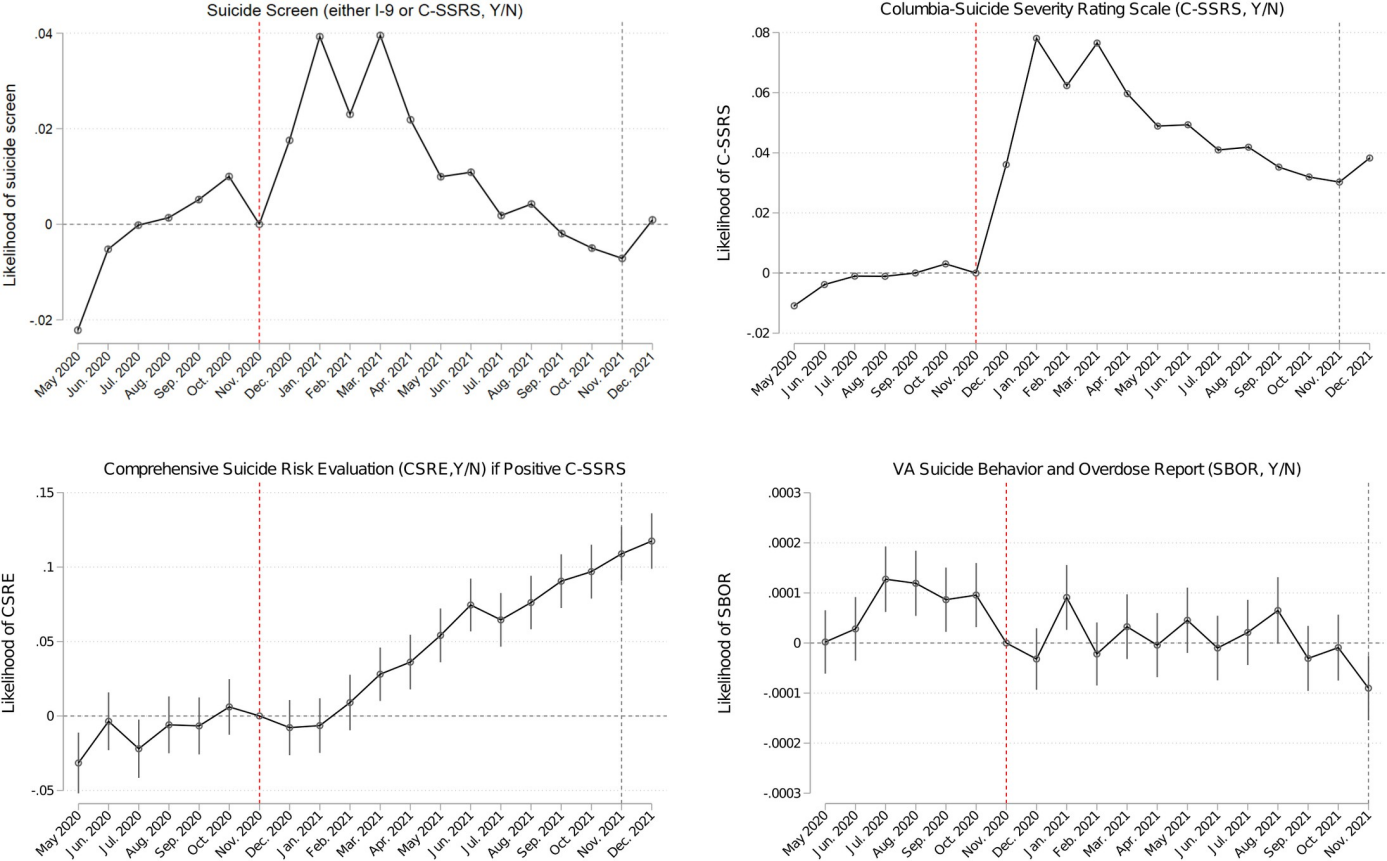
Model-adjusted outcome differences compared to Nov. 2020 –monthly coefficients from regression analyses. Note: Analyses not conditioned on positive C-SSRSs included 1,654,180 Veterans and 33,083,600 Veteran-monthly observations. Analyses conditioned on positive C-SSRSs included 57,673 Veterans and 85,237 Veteran-monthly observations. All models adjusted for Veterans’ age, sex, race, ethnicity, rurality of residence, number of physical and mental health chronic conditions, diagnoses of substance use disorder, post-traumatic stress disorder and depression, Nosos score, VA priority-based enrollment, marital status, homelessness indicator, high suicide risk indicator, cumulative monthly COVID-19 cases in the patient’s county. All models included indicators for patients’ closest facility to control for any time-invariant facility characteristics. In sensitivity analyses, models also adjusted for broadband coverage in patients’ residential zip-codes.

Figs 1 and 2 present results for the person-month-level analyses. Fig 1 presents the unadjusted average monthly likelihood of being screened via a C-SSRS and the monthly likelihood of being screened via either a C-SSRS or the historic I-9. Prior to November 2020, there was a differential in receipt of suicide screens between rural and urban Veterans, which narrowed post-November 2020.

Fig 2 presents the adjusted monthly likelihood of outcomes for the entire study cohort, compared to the month before implementation of universal screening. The monthly likelihood of being screened via the C-SSRS in the 6-months post-implementation ranged from 0.036 (95%CI: 0.036: 0.037) to 0.078 (0.077: 0.079) and via any suicide screen (either C-SSRS or I-9) ranged from 0.010 (0.009: 0.011) to 0.040 (0.039: 0.040). The average adjusted likelihood of receiving the C-SSRS and receiving any suicide screen in the 12 months post-universal screening implementation period compared to the pre-universal screening was 0.0539 (0.0536: 0.0541) and 0.0182 (0.0179: 0.0185), respectively (S2 Table). There were an additional 89,160 Veterans per month being screened for suicide via the C-SSRS and an additional 30,106 Veterans per month being screened via either the C-SSRS or I-9, compared to pre-universal screening (monthly regression coefficients indicating average increase in likelihood of receiving the C-SSRS were multiplied by the size of the cohort, 0.0539x1,654,180 = 89,160; 0.0182x1,654,180 = 30,106).

**Fig 3:**
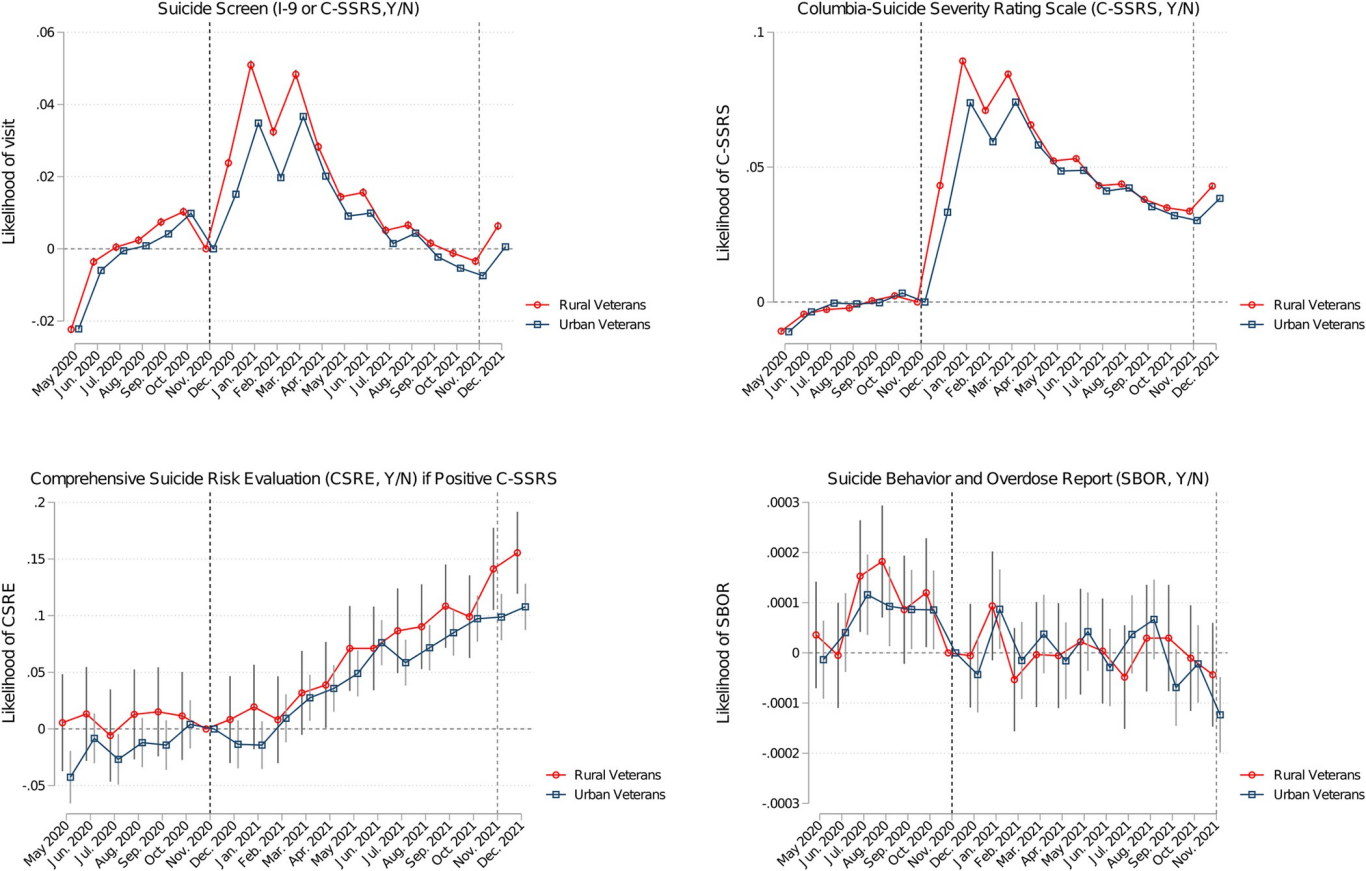
Model-adjusted outcome differences compared to Nov. 2020, for rural and urban Veterans–monthly coefficients from regression analyses. Note: Rural (urban) cohort analyses not conditioned on positive C-SSRSs included 485,592 (1,168,588) Veterans and 9,711,840 (23,371,760) Veteran-monthly observations. Rural (urban) cohort conditioned on positive C-SSRSs included 15,097 (42,576) Veterans and 21,040 (64,197) Veteran-monthly observations. All models adjusted for Veterans’ age, sex, race, ethnicity, number of physical and mental health chronic conditions, diagnoses of substance use disorder, post-traumatic stress disorder and depression, nosos score, VA priority-based enrollment, marital status, homelessness indicator, high suicide risk indicator, cumulative monthly COVID-19 cases in the patient’s county. All models included indicators for patients’ closest facility to control for any time-invariant facility characteristics. In sensitivity analyses, models also adjusted for broadband coverage in patients’ residential zip-codes.

Fig 3 presents the model-adjusted monthly likelihood of outcomes for the sub-cohort of rural Veterans and sub-cohort of urban Veterans. The average adjusted difference for rural and urban Veterans’ likelihood of being screened via C-SSRS during the 12-month period post-universal screening implementation was 0.0159/month (0.0153: 0.0166) (S2 Table). This translates to approximately 7,720/month (1.59% of 485,592) additional rural Veterans being screened via the C-SSRS during the year than their urban counterparts.

We conducted sensitivity analyses assessing if broadband coverage was driving the results observed for 97% of our cohort for whom broadband coverage data was available. The results after adjusting for broadband coverage were identical to the main results presented.

## Discussion

Efforts to prevent suicides nationwide have recently led to some universal suicide screening interventions [12–15], but these interventions have been relatively small-scale such as pilot programs within rural primary care practices or at individual hospital systems. To the best of our knowledge, the VA’s universal screening and evaluation requirements via Risk ID implemented November 2020 represents the largest suicide screening and evaluation intervention in the U.S., and this is the first study to evaluate VA’s Risk ID following the universal screening requirement. These efforts were specifically focused on VA patients engaged in mental health care and on examining differences in suicide screening outcomes for rural and urban patients.

In person-level analyses of our cohort of Veteran patients with mental health care needs, we found that 12 months post-universal screening and evaluation implementation, 1.3 million Veterans or 80% of the study cohort were screened or evaluated for suicide using either the I-9, C-SSRS or CSRE (Table 2). Among Veterans with positive screens, 80% received follow-up CSREs (Table 2). It is promising to see that 80% of our study cohort, a cohort with prior indications of mental health needs, was screened or evaluated post-universal screening implementation. Of note, the screens or evaluations could have occurred in mental health or other care settings. The 20% who were not screened likely included some patients who used VA care in 2019 but did not use any care in the post-universal screening implementation period or were not due in the post-universal screening implementation period we examined. In exploratory sub-analysis of Veterans with at least one mental health visit in the post-universal screening period, we found 91% of this sub-cohort was screened (in mental health settings or otherwise). Among the 9% of Veterans who had at least one mental health visit but were not screened, some patients may have not been due for an annual screen at the time of their mental health visit but it is also possible that some patients were missed due to gaps in implementation. Future work which uses data on visits across all settings (mental health and otherwise) would be helpful for understanding program implementation gaps at a more granular level. Finally, our finding that rural Veterans who had a mental health visit were more likely to have been screened than urban Veterans who had a mental health visit (S3 Table) may suggest the possibility that rural Veterans were more likely to be screened outside of mental health care settings, and this potential benefit of universal screening for rural Veterans should be further examined.

We also found that 20% of Veterans in our study cohort were screened or evaluated in the post-universal screening period even though they did not have a mental health visit during this period, which indicates a key strength of the universal screening requirement–that Veterans with indicated mental health care needs were getting screened/evaluated outside of mental health care settings. It is possible that greater than 20% of our study cohort was screened/evaluated outside of mental health settings, but as we did not specifically examine the settings where screens were administered for our analyses, additional studies examining settings where screens/evaluations were administered would be helpful. Furthermore, our finding that rural Veterans who did not have a mental health visit were more likely to have received suicide screens/evaluations (S3 Table) suggests the possibility that rural Veterans were more likely than urban Veterans to be screened/evaluated outside of mental health settings.

Next, in our adjusted person-month analyses, we found that after adjusting for differences in patient characteristics, VA’s universal screening/evaluation was associated with increased likelihood of being screened/evaluated for suicide. That is, patients who had previously sought VA mental health care had higher odds of getting screened/evaluated for suicide post-universal screening implementation compared to pre-universal screening implementation. The increase in suicide screens (via either historic I-9 or C-SSRS) in the 6 months post-universal screening implementation was driven by increased use of the C-SSRS screener. Gradually though, while the adjusted likelihood of receiving a C-SSRS remained higher than pre-universal screening, the likelihood of suicide screens (either I-9 or C-SSRS) returned to pre-universal screening levels as the C-SSRS appeared to substitute for the historic I-9 as intended by VA (Figs 1 and 2). Higher odds of getting screened for suicide indicated that patients’ mental health care had the potential to be augmented to address risk. This finding is especially important as VA’s suicide risk screening in ambulatory care prior to November 2020 was focused on Veterans who were due for annual depression and/or PTSD screening. Since a significant number of Veterans engaged in mental health care already have one or both of these diagnoses, they would not have been included in the screening processes and as a result would not have received the primary suicide screen despite being at increased risk for suicide.

Additionally, our adjusted person-month analyses demonstrated that universal screening implementation-associated increases in suicide screens/evaluation were greater for rural Veterans compared to urban Veterans. These findings emphasize the potential of universal screening to reach rural Veterans, who generally have higher barriers to accessing care and are considered higher risk for suicide. These results may be driven by the fact that rural Veterans were less likely than urban Veterans to get screened at baseline (Table 2), perhaps due to lower access to mental health providers and higher stigma associated with mental health help-seeking in rural areas [13]. Universal screening likely lowered these barriers for Veterans in rural areas and led to increased likelihood of being screened/evaluated across both mental and non-mental health settings, as suggested by our exploratory sub-analyses (S3 Table). VA’s use of virtual care may have further contributed to leveling the field across rural and urban Veterans by increasing access to virtual care for rural Veterans [33]. Future studies should explore potential mechanisms by examining rural-urban differences in care settings (whether mental health or other) where screens/evaluations occurred and by determining whether rural Veterans were more likely to get screened/evaluated via telehealth modalities.

Adjusted analyses also demonstrated that VA’s universal screening was associated with an increase in follow-up CSRE, conditioned on a positive C-SSRS (Fig 2). Although the point estimates for rural Veterans were higher than for urban Veterans, these differences were not statistically significant (Fig 3), possibly because these analyses, conditioned on positive C-SSRSs, could only be conducted for 1% of the study cohort with positive C-SSRSs. Future studies should analyze follow-up suicide-related care in larger study cohorts to assess whether universal screening improved follow-up care for rural Veterans more than for urban Veterans. Nevertheless, the current findings provide some assurance that once identified at risk via the C-SSRS, rural Veterans receive at least as frequent follow-up evaluations as urban Veterans.

Finally, post-universal screening implementation, there appeared to be a one-period increase in January 2021 in the likelihood of SBORs which was indistinguishable from pre-universal screening trend, with subsequent months exhibiting reduced likelihood of SBORs, compared to the pre-universal screening (Fig 2). The point estimates for rural Veterans were lower post-universal screening implementation than those for urban Veterans, but these differences were not statistically significant (Fig 3). Since the observed program association with SBORs was not a sufficiently abrupt change from pre-to-post implementation, these associations may not be attributable to universal screening, and may reflect a general trend of declining SBORs over time. Given the low frequency of the suicide behavior reports as an outcome, these results may signal potential clinical benefits of the program that should be monitored and further analyzed in future work, especially using a control group that would allow disentangling temporal trends from program-related associations.

### Limitations

There are limitations of this descriptive study. One limitation was the lack of a control group that experienced the same temporal trends but did not experience this policy. As such, it is possible that the observed associations are due to temporal changes other than universal screening. Nevertheless, because we used an event study approach and adjusted for a rich set of covariates using VA electronic health records data, we can directly observe trends in covariate-adjusted outcomes 6 months prior to the program which provide important context for evaluating post-program effects [34]. For most outcomes, we observed a flat pre-program trend, followed by a marked and statistically significant change post-program, which signals attributability of observed associations to VA’s universal screening.

An important data limitation was that we used an existing person-month dataset developed for another VA project to gain early and timely insights about the VA universal screen requirement. As such, while our dataset can provide a broad overview of the patterns, well-suited for our analysis comparing pre-post trends to ascertain associations with the date of program implementation, we did not have visit-level across all VA care settings and therefore could not assess more granular program implementation details such as screen rates or missed screens per visit or by setting of care (whether mental health or other), especially within the 30-month window when screens are supposed to be administered. Additional studies utilizing visit-level data across all care settings are needed to understand more granular program implementation details.

Next, although we identified that in the 12 months post-universal screening implementation 5.1% of the study cohort had at least one positive suicide screen, COVID-19 pandemic-related changes in the health care system from 2019 and 2020 and VA’s transition from using the I-9 screener to using C-SSRS screener prevented direct comparison of the number of individuals newly identified to be at-risk in 2019 vs. those identified to be risk in the post-universal screening implementation period. This is an important area for future work for assessing the value of a universal screening system.

Next, while promising, the VA’s universal screening approach would not be expected to reach rural Veterans who do not access VA care at all as this approach administers suicide screens or evaluations only during visits to VA facilities. As such, continued efforts will be needed to improve access to care for Veterans in rural areas or Veterans who face other access barriers to ensure the benefits of universal suicide screening can be realized further.

Finally, the scope of this evaluation is limited as we focused specifically on Veterans already engaged in mental health care. As such, our findings may not be generalize to those without a history of such care. Patients previously unengaged in mental health may benefit even more from a universalized approach to suicide screening, warranting additional evaluations of VA’s large-scale universal screening program.

## Conclusion

VA’s implementation of the universal suicide risk screening requirement in November 2020, intended to increase early and accurate detection of suicide risk among all Veterans presenting for care, is one critical strategy towards reducing Veteran suicides. It is also the largest universal suicide risk screening program in the U.S. and can thus offer valuable insights for health systems nationwide seeking to prevent suicides. Early evidence from evaluating this program specifically for a higher-risk patient population previously engaged in VA mental health care suggests that a universal approach can supplement suicide prevention care for this population, leveraging opportunities to screen for suicide risk in all patient interactions within the health care system. Early findings suggest that compared to urban patients, a universal approach may be especially advantageous for rural patients who may have fewer health care interactions due to the higher barriers they face in accessing care, and in particular mental health care. Given this program’s reliance on VA visits, it will be important to ensure continued access to VA and track patients receiving VA-paid care in the community or outside VA. It is critical to continue to track VA’s universal screening program and build upon this early evidence to inform national suicide prevention strategies.

## Supporting information

S1 Fig VA Risk ID’s transition from a 3-step to 2-step approach. (TIF)

S2 Fig Average monthly likelihood of being screened pre-and post-universal screening, stratified by Black and white race, ethnicity, and age group. (TIF)

S1 Table Universal screening associations in months post-implementation. (DOCX)

S2 Table Average monthly universal screening associations across the 12-months post-universal screening implementation. (DOCX)

S3 Table Sociodemographic differences in patterns of screening or evaluation for Veterans who had and who did not have a mental health visit in the post-universal screening period. (DOCX)

S1 File Methods details. (DOCX)
