# Supplementary figures and images for: VA’s implementation of universal screening and evaluation for the suicide risk identification program in November 2020 –Implications for Veterans with prior mental health needs
Source: PLoS One. 2023 Apr 11;18(4):e0283633. doi: 10.1371/journal.pone.0283633 (PMC10089346; doi:10.1371/journal.pone.0283633)

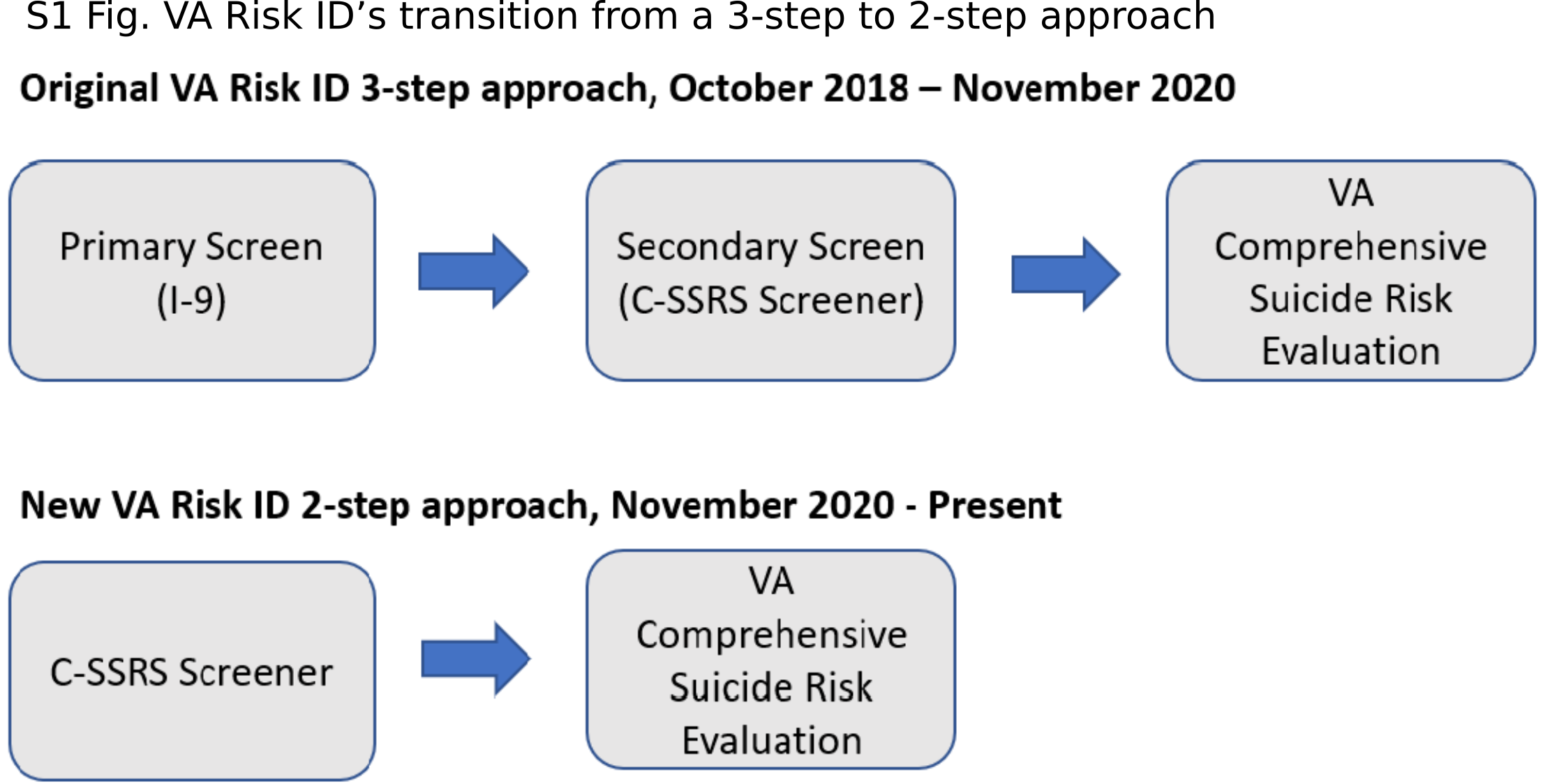

Supplement: S1 Fig — (TIF) [file pone.0283633.s001.tif]

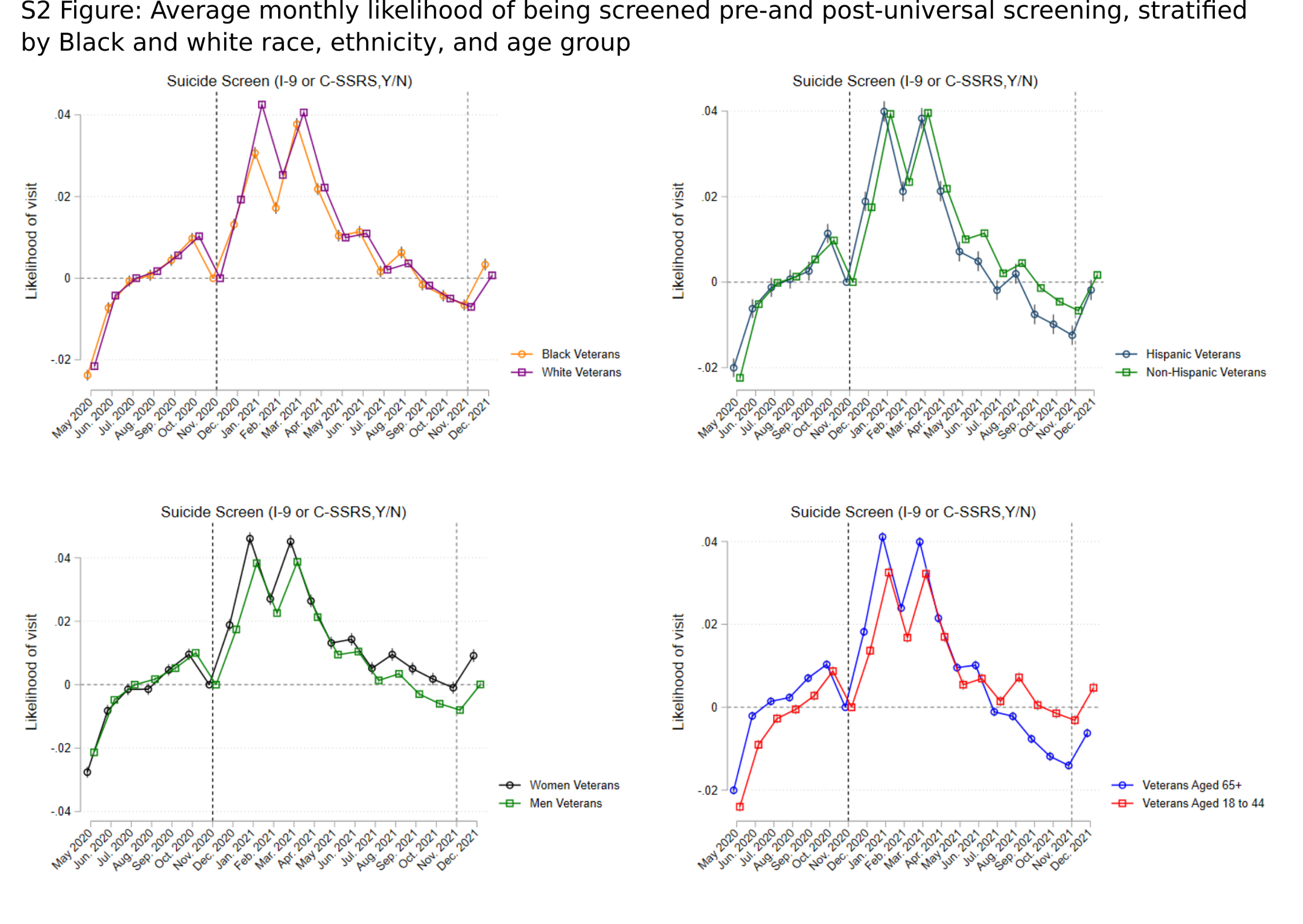

Supplement: S2 Fig — (TIF) [file pone.0283633.s002.tif]
