# Supplementary material for: VA’s implementation of universal screening and evaluation for the suicide risk identification program in November 2020 –Implications for Veterans with prior mental health needs
Source: PLoS One. 2023 Apr 11;18(4):e0283633. doi: 10.1371/journal.pone.0283633 (PMC10089346; doi:10.1371/journal.pone.0283633)
Supplement: S1 Table — (DOCX) [file pone.0283633.s003.docx]

**S1 Table: Universal Screening Associations in Months Post-Implementation**

| **Post-implementation coefficients from regression analyses** | | | | | |
| --- | --- | --- | --- | --- | --- |
|  | **C-SSRS** | **Suicide Screen (Either C-SSRS or Historic I-9)** | **CSRE if C-SSRS+** | **Suicide Risk Management visit if C-SSRS+** | **SBOR** |
| Dec. 2020 | 0.036  (0.036: 0.037) | 0.018  (0.017: 0.018) | -0.008  (-0.026: 0.011) | -0.007  (-0.019: 0.006) | -0.0000  (-0.0001: 0.0000) |
| Jan. 2021 | 0.078  (0.077: 0.079) | 0.039  (0.039: 0.040) | -0.006  (-0.025: 0.012) | -0.011  (-0.023: 0.001) | 0.0001  (0.0000: 0.0002) |
| Feb. 2021 | 0.062  (0.062: 0.063) | 0.023  (0.022: 0.024) | 0.009  (-0. 009: 0.028) | -0.010  (-0.022: 0.002) | -0.0000  (-0.0001: 0.0000) |
| Mar. 2021 | 0.077  (0.076: 0.077) | 0.040  (0.039: 0.040) | 0.028  (0.010: 0.046) | -0.013  (-0.025: 0.001) | 0.0000  (-0.0000: 0.0001) |
| Apr. 2021 | 0.060  (0.059: 0.060) | 0.022  (0.021: 0.023) | 0.036  (0.018: 0.055) | -0.006  (-0.018: 0.007) | -0.0000  (-0.0001: 0.0001) |
| May 2021 | 0.049  (0.048: 0.049) | 0.010  (0.009: 0.011) | 0.054  (0.036: 0.072) | 0.002  (-0.010: 0.015) | 0.0000  (-0.0000: 0.0001) |
| Jun. 2021 | 0.049  (0.049: 0.050) | 0.011  (0.010: 0.012) | 0.075  (0.057: 0.092) | 0.015  (0.002: 0.029) | -0.0000  (-0.0001: 0.0001) |
| Jul. 2021 | 0.041  (0.040: 0.042) | 0.002  (0.001: 0.003) | 0.065  (0.047: 0.083) | 0.010  (-0.003: 0.024) | 0.0000  (-0.0000: 0.0001) |
| Aug. 2021 | 0.042  (0.041: 0.042) | 0.004  (0.004: 0.005) | 0.076  (0.058: 0.094) | 0.004  (-0.010: 0.017) | 0.0001  (-0.000: 0.0001) |
| Sep. 2021 | 0.035  (0.035: 0.036) | -0.002  (-0.003: -0.001) | 0.091  (0.072: 0.109) | 0.015  (0.001: 0.028) | -0.0000  (-0.0001: 0.0000) |
| Oct. 2021 | 0.032  (0.031: 0.033) | -0.005  (-0.006: -0.004) | 0.097  (0.079: 0.115) | 0.035  (0.021: 0.049) | -0.0000  (-0.0001: 0.0001) |
| Nov. 2021 | 0.030  (0.030: 0.031) | -0.007  (-0.008: -0.006) | 0.109  (0.091: 0.127) | 0.043  (0.028: 0.057) | -0.0001  (-0.0002: -0.0000) |
| Dec. 2021 | 0.038  (0.038: 0.039) | 0.001  (0.000: 0.002) | 0.117  (0.099: 0.136) | 0.051  (0.036: 0.066) |  |

Note: All models adjusted for Veterans’ age, sex, race, ethnicity, rurality of residence, number of physical and mental health chronic conditions, diagnoses of substance use disorder, post-traumatic stress disorder and depression, nosos score, VA priority-based enrollment, marital status, homelessness indicator, high suicide risk indicator, cumulative monthly COVID-19 cases in the patient’s county. All models included indicators for patients’ closest facility to control for any time-invariant facility characteristics. In sensitivity analyses, models also adjusted for broadband coverage in patients’ residential zip-codes. Due to potential lags in the most recent suicide behavior data, we excluded December 2021 from the SBOR analysis.
