# Supplementary material for: VA’s implementation of universal screening and evaluation for the suicide risk identification program in November 2020 –Implications for Veterans with prior mental health needs
Source: PLoS One. 2023 Apr 11;18(4):e0283633. doi: 10.1371/journal.pone.0283633 (PMC10089346; doi:10.1371/journal.pone.0283633)
Supplement: S2 Table — (DOCX) [file pone.0283633.s004.docx]

**S2 Table: Average Monthly Universal Screening Associations across the 12-months post-Universal Screening**

| Average Association of Universal Screening with outcomes in the 12 Months Post-Universal Screening | | |
| --- | --- | --- |
|  | C-SSRS | Suicide Screen (Either C-SSRS or Historic I-9) |
| Post-RiskID | 0.0539  (0.0536: 0.0541) | 0.0182  (0.0179: 0.0185) |
| Average difference in associations for rural Veterans (vs. urban Veterans) in the 12 Months Post-Universal Screening | | |
| Rural x Post-RiskID | 0.0159  (0.0153: 0.0166) | 0.0190  (0.0183: 0.0198) |
