# Supplementary material for: VA’s implementation of universal screening and evaluation for the suicide risk identification program in November 2020 –Implications for Veterans with prior mental health needs
Source: PLoS One. 2023 Apr 11;18(4):e0283633. doi: 10.1371/journal.pone.0283633 (PMC10089346; doi:10.1371/journal.pone.0283633)
Supplement: S3 Table — (DOCX) [file pone.0283633.s005.docx]

**S3 Table: Sociodemographic Differences in Patterns of Screening or Evaluation for Veterans who had and who did not have a Mental Health Visit in the Post-Universal Screening Period**

| **Sub-Cohort with ≥1 Mental Health visit during Post-Universal Screening Period not Screened or Evaluated during this period (n=92,501; 5.6% of study cohort)** | | | | ***p-val.***^b^ |
| --- | --- | --- | --- | --- |
| **Rural** | 5.0% | **Urban** | 5.8% | <0.001 |
| **Black** | 5.5% | **White** | 5.6% | 0.020 |
| **Women** | 5.7% | **Men** | 5.6% | 0.004 |
| **Sub-Cohort with *no* Mental Health visit during Post-Universal Screening Period who were screened/evaluated during this period (n=329,773; 20% of the study cohort)** | | | | ***p-val.***^b^ |
| **Rural** | 21.3% | **Urban** | 19.4% | <0.001 |
| **Black** | 20.1% | **White** | 20.0% | 0.034 |
| **Women** | 16.7% | **Men** | 20.4% | <0.001 |

^a^ Differences in pre- vs. post- implementation percentages were tested using the two-sample t-test. P-values <0.05 were considered statistically significant.
