# Supplementary material for: VA’s implementation of universal screening and evaluation for the suicide risk identification program in November 2020 –Implications for Veterans with prior mental health needs
Source: PLoS One. 2023 Apr 11;18(4):e0283633. doi: 10.1371/journal.pone.0283633 (PMC10089346; doi:10.1371/journal.pone.0283633)
Supplement: S1 File — (DOCX) [file pone.0283633.s006.docx]

**S1 File. Methods Details**

We used a pre-post event study design to examine descriptively the associations between timing of VA’s universal implementation of the C-SSRS suicide screener and the likelihoods of Veterans receiving a suicide screen, a follow-up in-depth suicide evaluation if C-SSRS was positive, as well as the likelihood of a suicide behavior and overdose report. Event study specifications were as follows:

$$Y_{i,m}= \beta_{0}+{\beta_{1-19}\left( month relative to UniversalCSSRS \right)}_{m}+\beta_{20}{rural}_{i}+\beta_{21}{gender}_{i}+ \beta_{22}{race}_{i}+ \beta_{23}{ethnicity}_{i}+\beta_{24}{age}_{i}+\beta_{25}N{umMHconds}_{i} {+ \beta}_{26}{NumPHYSconds}_{i}+ \beta_{27}{Depression}_{i}+ \beta_{28}{PTSD}_{i}+ \beta_{29}{SUD}_{i}+ \beta_{30}{Nosos\_score}_{i}+\beta_{31}{VApriority}_{i}+ \beta_{32}{maritalstatus}_{i}+\beta_{33}{reachvetstatus}_{i}+\beta_{34}{cumltv covid cases in county}_{i,m}+{closestfacilityFEs}_{i}+ \varepsilon$$

where $Y_{i,m}$ is the outcome of interest, *i* denotes an individual, and m denotes months*.* $\beta_{1-19}$were the coefficients of interest (plotted in Figure 2) which reflect the associations with VA’s universal screening each month pre- and post-universal screening. Month relative to Universal CSSRS or the universal screen requirement was calculated as observation month minus November 2020 such that relative month = 0 is November 2020 when universal screening. These coefficients indicated differences in outcomes in each pre- and post- month, compared to the baseline or implementation month, November 2020.

For analyses stratified by rurality of residence, the same event study specification was used on a sub-cohort of rural Veterans and on a sub-cohort of urban Veterans (without the rural indicator). $\beta_{1-19}$were coefficients of interest (plotted in Figure 3).

In sensitivity analyses, models also adjusted for broadband coverage in patients’ residential zip-codes.
